# Editorial: Emerging issues in English language education with psycho-emotional traits in the spotlight: futurology in language studies

**DOI:** 10.3389/fpsyg.2026.1853075

**Published:** 2026-05-14

**Authors:** Hamed Barjesteh, Samantha Curle, Neda Fatehi Rad, Deyuan He, Mehdi Manoochehrzadeh

**Affiliations:** 1 Department of English Language and Literature, Am.C., Islamic Azad University, Amol, Iran; 2 Department of Education, University of Bath, Bath, United Kingdom; 3 Department of English Language and Literature, Khazar University, Baku, Azerbaijan; 4 Department of English Language, Ke.C., Islamic Azad University, Kerman, Iran; 5 Faculty of Arts and Social Sciences, Universiti Brunei Darussalam, Bandar Seri Begawan, Brunei; 6 Zerodale Inc. Centre for Research in Entrepreneurship Education and Development, Toronto, ON, Canada

**Keywords:** emotional intelligence, futurology in language studies, motivation, psycho-emotional traits, teacher wellbeing

English language education is increasingly shaped by empirically grounded insights from cognitive science, affective psychology, and educational innovation, particularly research demonstrating systematic links between affective variables, learning processes, and educational outcomes in multilingual and EMI contexts (e.g., Dörnyei and Ryan, 2015; MacIntyre et al., 2019). Research over the past decade has made clear that psycho-emotional traits are not peripheral to language achievement; they are central to how learners engage, persist, and construct meaning in multilingual contexts (Dewaele, 2019; Solhi et al., 2024). ^1^ This Research Topic places psycho-emotional factors at the forefront of inquiry and examines their implications for the future of language education. Across diverse empirical and theoretical contributions, the Research Topic foregrounds a constellation of psycho-emotional constructs, including language anxiety, enjoyment, resilience, academic buoyancy, emotion regulation, self-efficacy, engagement, and wellbeing. Rather than treating these variables as discrete traits, the contributions conceptualize them as dynamically interrelated systems operating across cognitive, motivational, and sociocultural dimensions of language learning. These variables are examined not as isolated attributes, but as dynamic processes that interact with cognition, motivation, social belonging, and instructional design. Emotional and psychological traits shape how learners respond to academic pressure, navigate uncertainty, and sustain effort in demanding learning environments. Likewise, teachers' psycho-emotional characteristics influence instructional quality, classroom climate, and professional development trajectories.

A central contribution of this Research Topic lies in its forward-looking orientation. While much prior research has relied on retrospective analyses of affective variables, the present Research Topic adopts a prospective perspective. Within this Research Topic, futurology in language studies is operationalized as a form of anticipatory inquiry that examines how psycho-emotional traits may shape the long-term sustainability, adaptability, and ethical orientation of language education systems under conditions of globalization, digitalization, and policy intensification. Rather than merely documenting current challenges, the articles consider how educators and learners can consciously cultivate resilience, adaptability, emotional intelligence, and mindfulness as competencies for an evolving global landscape.

The intersection of psycho-emotional traits and pedagogical practice is explored across multiple domains, including digital learning environments, teacher education, curriculum development, assessment, intercultural communication, and innovative instructional models. Interventions such as mindfulness practices, social–emotional learning frameworks, and emotional intelligence training are examined for their potential to enhance both well-being and linguistic performance. In this way, psycho-emotional development is positioned not only as supportive of language acquisition but as integral to educational effectiveness and sustainability.

Importantly, this Research Topic acknowledges that the influence of psycho-emotional characteristics on the long-term quality of teaching and learning remains an open and evolving question. By integrating quantitative, qualitative, and mixed-methods approaches, the contributions advance a more comprehensive understanding of how affective and psychological variables shape present experiences and future trajectories in English language education. Taken together, the articles in this Research Topic map a significant research frontier at the nexus of psychology and language studies. They call for a holistic and strategically informed approach that recognizes emotional and psychological preparedness as foundational to both academic success and sustainable professional growth. It is hoped that this Research Topic will stimulate continued scholarship and practical innovation to foster psychologically informed, future-oriented language education. Across various research designs for the current topical issue, the contributions converge on a core insight: affective and cognitive processes are reciprocally constitutive rather than hierarchically ordered. This Research Topic brings together contributions that collectively advance understanding across the following thematic and methodological domains

1. *Exploring the roles of self-actualization, language self-efficacy, and academic emotions in EMI students' achievement* (Guan et al.)

This large-scale quantitative study investigates how self-actualization and language self-efficacy shape academic achievement among university students enrolled in English Medium Instruction (EMI) programmes in China, with internal locus of control and learning anxiety modeled as mediating variables. Using survey data from 480 students and Structural Equation Modeling, the authors demonstrate that self-actualization indirectly enhances academic achievement primarily through strengthening students' internal locus of control, while language self-efficacy exerts both direct positive effects on achievement and indirect effects through reduced learning anxiety. Notably, internal locus of control emerges as the strongest mediator in the model. The study advances EMI research by empirically challenging linguistic deficit framings and demonstrating how humanistic and motivational constructs operate as mediating mechanisms between instructional context and academic achievement. Its contribution lies in offering a theoretically integrated, empirically robust account of how psycho-emotional resources underpin sustainable achievement in EMI contexts.

2. *Change in perspective, change in educational policy* (Reize et al.)

This policy-focused action research study examines how a sustained shift in pedagogical perspective led to systemic departmental reform in EFL teacher education, with a particular emphasis on addressing foreign language anxiety and emotional safety. Drawing on multiple cycles of action research and informed by expansive learning theory, the authors document the implementation of the ACCESSES framework, integrating autonomy, collaboration, communication, empowerment, scaffolding, and emotional safety. Findings demonstrate that student teachers' oral performance was shaped more by social and emotional factors than by linguistic knowledge alone, prompting a reconceptualization of assessment, curriculum design, and professional collaboration. The study's significance lies in its demonstration of how emotion-sensitive pedagogy can be institutionalized through policy and collective practice, offering a scalable model for embedding psycho-emotional awareness into teacher education and language policy reform.

3. *The tone of teachers' melody and wellbeing in digital space* (Mirzapour et al.)

This phenomenological study explores how the integration of digital technologies shapes EFL teachers' physical and social wellbeing in online and hybrid teaching contexts. Based on in-depth interviews and narrative data from experienced teachers in Iran, the analysis reveals a complex duality. Digital tools facilitate efficiency, cognitive stimulation, and global connectivity, yet simultaneously contribute to exhaustion, health risks, reduced face-to-face interaction, and strained social relationships. Grounded in positive psychology and digital wellbeing frameworks, the study foregrounds teachers' emotional labor and embodied experiences of digital teaching. Its contribution lies in shifting attention from student-centered digital outcomes to teacher wellbeing as a central condition for sustainable language education, highlighting the need for institutional guidance that supports healthy, reflective, and ethically informed technology use.

4. *Balancing quality and well-being: a study on the impact of quality assurance measures on burnout among EFL teachers in Vietnam* (Tran et al.)

This qualitative phenomenological study investigates how intensified quality assurance regimes in Vietnamese higher education affect EFL teachers' wellbeing, with burnout examined through the lenses of emotional exhaustion, depersonalization, and reduced professional accomplishment. Drawing on semi-structured interviews with teachers across institutional contexts, the findings reveal that QA measures significantly increase administrative workload, heighten performance pressures, and erode teachers' sense of professional autonomy. While participants reported coping strategies aimed at preserving commitment and fulfillment, the study demonstrates that unchecked QA demands pose serious risks to teacher wellbeing and pedagogical sustainability. The article makes a strong contribution by problematizing quality discourses that prioritize metrics over human capacity, and by positioning teacher wellbeing as a prerequisite for genuine educational quality in EFL and EMI-adjacent systems.

5. *Developmental trajectories of and reciprocal relationships between Chinese university students' foreign language reading self-efficacy and reading strategy use* (Zhou and Zhang)

In this longitudinal mixed-methods study, Zhou and Zhang examine the developmental trajectories and reciprocal relationships between Chinese university students' foreign language reading self-efficacy and reading strategy use. Tracking 293 EFL undergraduates across five time points, the authors employ parallel latent growth modeling and cross-lagged analyses. Findings reveal fluctuating yet overall increasing self-efficacy, steadily rising strategy use, and a bidirectional predictive relationship, with strategy use exerting a stronger influence on self-efficacy. The study highlights the dynamic interplay between affective and cognitive factors in EFL reading development.

6. *Quantum psychology approach on enjoyment as mediator in relationship between L2 flow and engagement* (Çelik)

Çelik applies a quantum psychology perspective to examine whether enjoyment mediates the relationship between L2 psychological flow and academic engagement among 162 Turkish high school EFL learners in AI-assisted classrooms. Using path analysis, results show that flow does not directly predict engagement; instead, flow significantly increases enjoyment, which in turn enhances engagement, indicating full mediation. The findings challenge linear SLA models and suggest that affective states, particularly enjoyment, probabilistically shape learner engagement in technology-enhanced language learning contexts.

7. *How social–emotional learning promotes reading achievement? A systematic review of mechanisms and instructional design* (Hua et al.)

In this PRISMA-guided systematic review of 31 empirical studies, Hua et al. examine how Social–Emotional Learning (SEL) enhances students' reading achievement. The synthesis identifies four primary pathways: emotional regulation, intrinsic motivation and engagement, peer-supported social interaction, and cognitive/metacognitive development. SEL improves reading comprehension, vocabulary acquisition, and long-term literacy behaviors. The review argues that SEL should be embedded as a foundational component of reading instruction rather than treated as a supplementary program, with implications for curriculum design, teacher training, and educational policy reform.

8. *Cognitive and affective processes in second language oral communication: a mixed methods research* (Portugal-Toro et al.)

Using a mixed-methods approach, the article by Portugal-Toro et al. conducted a study on cognitive and affective processes in second-language oral communication. To be more specific, this article offers a distinct contribution to English language teaching by integrating multiple variables that influence students' ability to communicate effectively in English, offering insights that may inform more holistic approaches to developing students' communicative skills. These findings contribute to ongoing debates on the interdependence of affective and cognitive processes in oral communication, with implications for pedagogical design that foregrounds emotional regulation alongside linguistic development.

9. *Mechanisms of foreign language learning anxiety and enhancement strategies among Chinese tertiary students: a grounded theory approach* (Gao and Zuo)

Gao and Zuo's article focuses on foreign language learning anxiety and the enhancement strategies used by Chinese tertiary students. To alleviate this anxiety, they propose teaching strategies grounded in positive psychology, including enhancing self-regulation, instilling a growth mindset, fostering flow experiences, building a positive self-concept, and fostering a pleasurable classroom atmosphere. They recommended that future research should adopt a dynamic complexity theory perspective to explore the trends in anxiety emotions and their interrelations with other affective factors, aiming to develop more effective intervention measures.

10. *Effects of perceived English teacher support on student engagement among Chinese EFL undergraduates: L2 motivational self system as the mediator* [Zhang and Hu (a)]

Based on the perspective of the second language motivational self-system theory, Zhang and Hu (a)'s article used a structural equation model to explore the general profile of 2,633 Chinese undergraduates of English as a foreign language perceived teacher support, second language (L2) motivation, and student engagement in English classrooms, and the interrelationships among the three variables. Their results showed that teacher support and student engagement were high, and L2 motivation was medium-high in English learning. They also argued that perceived teacher support significantly correlated with L2 motivation and profoundly influenced student engagement. Moreover, they found that the L2 motivational self-system mediated the effect of perceived teacher support on student engagement, and that L2 learning experience exerted a greater mediating effect than the ideal L2 self and ought-to L2 self. Their findings deepen understanding of the interaction mechanism among these variables and provide useful references to improve student engagement and learning effectiveness.

11. *The relationship between perceived teacher support and student engagement in Chinese senior high school English classrooms: the mediating role of learning motivation* [Zhang and Hu (b)]

In a structural equation modeling study of 314 Chinese senior high school students, Zhang and Hu (b) investigate how perceived teacher support influences student engagement in English classrooms, with learning motivation as a mediator. Results show that teacher support positively predicts engagement both directly and indirectly. Learning motivation partially mediates this relationship, with intrinsic motivation exerting a stronger mediating effect than extrinsic motivation. The findings highlight the critical role of autonomy, emotional, and cognitive teacher support in fostering motivated and engaged English learners.

12. *Investigating EFL students' engagement in project-based speaking activities: from a multi-dimensional perspective* (Zhong et al.)

In a quasi-experimental mixed-methods study, Zhong et al. examine the impact of Project-Based Learning (PBL) on multidimensional engagement in Chinese EFL speaking classes. Ninety-six first-year polytechnic students were assigned to either a PBL group or a conventional teaching method (CTM) group over 14 weeks. MANOVA results show that PBL significantly enhances behavioral, emotional, and cognitive engagement, but not agentic engagement. Interviews reveal increased interest, focused effort, strategic thinking, and proactive participation. While PBL fosters deeper engagement overall, limited gains in agentic engagement suggest the need for greater scaffolding and autonomy support in EFL classrooms.

13. *Beyond standard English: how Global Englishes enhances creativity, reduces language anxiety, and increases willingness to communicate* (Budiman and Liu)

In the perspective article, Budiman and Liu propose a conceptual framework linking Global Englishes Language Teaching (GELT) to key psychological outcomes in second language acquisition. Moving beyond native-speaker norms, GELT validates diverse English varieties, reframes errors as communicative resources, and promotes multilingualism. The authors argue that this inclusive paradigm enhances learner creativity, reduces language anxiety, and increases willingness to communicate (WTC). By shifting emphasis from accuracy to intelligibility and agency, GELT fosters confidence, resilience, and communicative autonomy. The framework offers theoretical and pedagogical implications for designing psychologically supportive, learner-centered English classrooms in multilingual global contexts.

14. *The impact of chatbots on emotional intelligence in AI-assisted EFL writing* (Kazazoglu and Turun Özel)

Kazazoglu and Turun Özel investigate the impact of chatbot-assisted EFL writing on the emotional intelligence (EI) of 44 pre-service English teachers in Türkiye. Using a quasi-experimental pre-test–post-test design and the Turkish adaptation of the Bar-On EQ-i, participants engaged in a 4-week AI-supported creative writing program. Results revealed no significant overall change in EI, though adaptability showed a slight, non-robust increase. Notably, higher AI competence and technology engagement were moderately negatively correlated with certain EI dimensions. The findings underscore the complex, context-dependent relationship between AI integration and emotional development in language education.

## Converging trajectories across the Research Topic

Synthesizing the contributions, three analytically distinct yet interconnected trajectories emerge across the Research Topic: (a) motivational and self-system architectures, (b) the emotional ecology of teaching within institutional systems, and (c) technology-mediated learning and emotional adaptation. Several studies demonstrate how internal psychological systems mediate educational outcomes. Research on EMI students reveals that self-actualization and language self-efficacy indirectly shape achievement through internal locus of control and anxiety regulation. Structural equation models examining teacher support show consistent pathways through the L2 motivational self-system and intrinsic motivation to engagement. Longitudinal modeling of reading self-efficacy and strategy use highlights bidirectional reinforcement between affective belief systems and cognitive behavior. Collectively, these findings reinforce the view that motivation is not a static trait but a dynamic psychological ecosystem, continuously reshaped by instructional context, perceived support, and self-regulatory processes.

A second trajectory centers on teacher wellbeing and institutional pressures. Phenomenological investigations of digital teaching environments reveal dualities: cognitive stimulation and global connectivity coexist with exhaustion, emotional labor, and health strain. Studies on quality assurance regimes document burnout risks associated with metric-driven accountability systems. Together, these contributions challenge technocratic narratives of educational efficiency and underscore a foundational psychological principle: educational quality cannot be disentangled from human wellbeing. Sustainable language education requires emotional sustainability in teachers as much as in learners.

The third trajectory critically engages with AI-mediated learning environments. Project-based learning, Global Englishes pedagogy, and SEL-informed instructional models show that psychologically inclusive approaches enhance engagement, creativity, and willingness to communicate. However, findings from AI-assisted writing research introduce a necessary complexity: while adaptability may increase in chatbot-supported contexts, higher AI competence and technology engagement correlate negatively with certain emotional intelligence dimensions. These findings complicate techno-optimistic assumptions by demonstrating that AI-mediated learning environments may simultaneously support adaptability while constraining certain dimensions of emotional and interpersonal development. It suggests that AI integration can simultaneously scaffold adaptability and risk-attenuating interpersonal emotional competencies if not pedagogically balanced. Human–AI interaction in language learning thus emerges as a psychologically ambivalent frontier requiring ethical and instructional safeguards.

Implications for Educational Psychology

For educational psychology, this Research Topic advances three critical propositions:

- Psycho-emotional variables must be treated as structural constructs within language education models, not supplementary correlates.
- Teacher emotional wellbeing constitutes a foundational condition for sustainable instructional quality.
- AI-mediated learning requires psychologically informed design to prevent unintended erosion of interpersonal and stress-regulation capacities.

The issue also highlights methodological maturation in the field, including the use of structural equation modeling, latent growth modeling, grounded theory approaches, and PRISMA-guided synthesis. Yet it also signals future directions: ecological momentary assessment, multilevel modeling, physiological indicators of stress and engagement, and longitudinal tracking of AI-emotion interaction represent promising next steps.

## Reframing the future of language education

A central conclusion of this Research Topic is that language education can no longer treat psycho-emotional processes as peripheral variables, but must instead conceptualize them as constitutive elements of learning, teaching, and institutional design. In globalized, digitized, and accountability-driven systems, psycho-emotional competencies determine not only immediate achievement but also long-term adaptability, professional sustainability, and ethical human development. By mapping intersections between emotional intelligence, motivation, policy reform, digital wellbeing, social–emotional learning, AI integration, and Global Englishes paradigms, this Research Topic delineates a psychological frontier in language studies. It calls for a holistic model in which emotional preparedness, motivational architecture, and institutional ecology are treated as coequal dimensions of educational design. The future of English language education will not be secured by curricular innovation alone. It will depend on the deliberate cultivation of psychologically resilient learners and emotionally supported teachers capable of navigating uncertainty, technological transformation, and intercultural complexity.

## Footnotes

1 Wang, Y. (2016). *Chinese Students' Emotional Experiences in Learning English as a Foreign Language* (Ph.D. Dissertation). University of Texas at Austin.
